# Supplementary material for: Physical activity prevalence and associated factors among Zimbabwean undergraduate students: A cross-sectional study
Source: PLOS Glob Public Health. 2025 Jul 9;5(7):e0004866. doi: 10.1371/journal.pgph.0004866 (PMC12240316; doi:10.1371/journal.pgph.0004866)
Supplement: S1 Table — (DOCX) [file pgph.0004866.s001.docx]

**Supporting information**

### **S1 Table: IPAQ summative indices**

| **Physical activity category** | **Median (Q_1_ – Q_3_)** |
| --- | --- |
| **Vigorous** | 0.0 (0.0 - 1200.0) |
| **Moderate** | 0.0 (0.0 - 720.0) |
| **Light** | 990.0 (396.0 – 2425.5) |
| **Sedentary** | 300.0 (180.0- 422.0) |
