# Supplementary material for: Physical activity prevalence and associated factors among Zimbabwean undergraduate students: A cross-sectional study
Source: PLOS Glob Public Health. 2025 Jul 9;5(7):e0004866. doi: 10.1371/journal.pgph.0004866 (PMC12240316; doi:10.1371/journal.pgph.0004866)
Supplement: S2 Table — (DOCX) [file pgph.0004866.s002.docx]

### **S2 Table: EBBS frequencies**

| **Domain** | **Question items** | **Strongly disagree, n (%)** | **Disagree, n (%)** | **Agree, n (%)** | **Strongly agree, n (%)** |
| --- | --- | --- | --- | --- | --- |
| Barriers |  |  |  |  |  |
| Facilities access (FA) | It costs too much to exercise | 193(15.9) | 717(58.9) | 220(18.1) | 87(7.1) |
|  | Exercise facilities do not have convenient schedules for me | 56(4.6) | 478(39.3) | 507(41.7) | 176(14.5) |
|  | I think people in exercise clothes look funny | 254(20.9) | 677(55.6) | 196(16.1) | 90(7.4) |
|  | There are too few places for me to exercise | 75(6.2) | 421(34.6) | 554(45.5) | 167(13.7) |
| Time Expenditure (TE) | Exercising takes too much of my time | 100(8.2) | 270(22.2) | 727(59.7) | 120(9.9) |
|  | Exercise takes too much time from my family | 206(16.9) | 745(61.2) | 171(14.1) | 95(7.8) |
|  | Exercise takes too much time from my family responsibilities | 189(15.5) | 708(58.2) | 236(19.4) | 84(6.9) |
| Benefits |  |  |  |  |  |
| Life Enhancement (LE) | Exercising helps me sleep better at night | 32(2.6) | 155(12.7) | 691(56.8) | 339(27.9) |
|  | Exercise helps me decrease fatigue | 47(3.9) | 269(22.1) | 697(57.3) | 204(16.8) |
|  | Exercising increases my mental alertness | 13(1.1) | 78(6.4) | 751(61.7) | 375(30.8) |
|  | Exercising allows me to carry out normal activities without becoming tired. | 22(1.8) | 189(15.5) | 677(55.6) | 329(27.0) |
| Physical Performance (PE) | Exercising increases my muscle strength | 17(1.4) | 49(4.0) | 708(58.2) | 443(36.4) |
|  | Exercising increases my level of physical fitness | 10(0.8) | 26(2.1) | 639(52.5) | 542(44.5) |
|  | My muscle tone is improved by exercise | 10(0.8) | 91(7.5) | 771(63.4) | 345(28.3) |
|  | Exercise improves the functioning of my cardiovascular system | 10(0.8) | 36(3.0) | 695(57.1) | 476(39.1) |
|  | Exercising increases my stamina | 8(0.7) | 77(6.3) | 714(58.7) | 418(34.3) |
|  | Exercise improves overall body functioning for me | 5(0.4) | 59(4.8) | 778(63.9) | 375(30.8) |
|  | Exercising improves the way my body looks | 13(1.1) | 49(4.0) | 759(62.4) | 396(32.5) |
| Psychological Outlook (PO) | I enjoy exercise | 38(3.1) | 153(12.6) | 648(53.2) | 378(31.1) |
|  | Exercise decreases feelings of stress and tension for me | 22(1.8) | 103(8.5) | 632(51.9) | 460(37.8) |
|  | Exercise improves my mental health | 23(1.9) | 72(5.9) | 627(51.5) | 495(40.7) |
|  | Exercising makes me feel relaxed | 30(2.5) | 228(18.7) | 672(55.2) | 287(23.6) |
| Social Interaction (SI) | Exercising lets me have contact with persons and friends I enjoy | 44(3.6) | 372(30.6) | 570(46.8) | 231(19.0) |
|  | Exercising is a good way for me to meet new people | 34(2.8) | 251(20.6) | 709(58.3) | 223(18.3) |
|  | Exercising is good entertainment for me | 54(4.40 | 263(21.6) | 659(54.1) | 241(19.8) |
|  | Exercising increases my acceptance by others | 80(6.6) | 388(31.9) | 583(47.9) | 166(13.6) |
|  |  |  |  |  |  |
