# Supplementary material for: Physical activity prevalence and associated factors among Zimbabwean undergraduate students: A cross-sectional study
Source: PLOS Glob Public Health. 2025 Jul 9;5(7):e0004866. doi: 10.1371/journal.pgph.0004866 (PMC12240316; doi:10.1371/journal.pgph.0004866)
Supplement: S3 Table — (DOCX) [file pgph.0004866.s003.docx]

### **S3 Table: EBBS summative indices**

| **Variable** | **Mean, SD** | **Median, IQR** | **Range** |  |
| --- | --- | --- | --- | --- |
|  |  |  | **Minimum** | **Maximum** |
| **Benefits** |  |  |  |  |
| Life Enhancement (LE) | 12.3(2.1) | 12.0(11.0 – 14.0) | 4 | 16 |
| Physical Performance (PP) | 23.0(2.9) | 22.0(21.0 – 23.0) | 7 | 28 |
| Psychological Outlook (PO) | 12.7(2.2) | 13.0(12.0 – 14.0) | 4 | 16 |
| Social Interaction (SI) | 11.3(2.3) | 11.0(10 – 13.0) | 4 | 16 |
| Subscale total | 59.3(7.9) | 58.0(55.0 – 65.0) | 19 | 76 |
| **Barriers** |  |  |  |  |
| Facilities access (FA) | 9.6(2.1) | 10.0(8.0 – 11.0) | 4 | 16 |
| Time Expenditure (TE) | 7.0(1.3) | 7.0(6.0 – 8.0) | 3 | 12 |
| Subscale Total | 16.6(2.7) | 16.0(15.0 – 18.0) | 7 | 26 |
