# Supplementary material for: Physical activity prevalence and associated factors among Zimbabwean undergraduate students: A cross-sectional study
Source: PLOS Glob Public Health. 2025 Jul 9;5(7):e0004866. doi: 10.1371/journal.pgph.0004866 (PMC12240316; doi:10.1371/journal.pgph.0004866)
Supplement: S4 Table — (DOCX) [file pgph.0004866.s004.docx]

### **S4 Table: KAP summative indices**

| **Variable** | **Mean (SD)** | **Range [Min- max]** |
| --- | --- | --- |
| Knowledge subscore | 27 (3.6) | 17 [7 -24] |
| Attitudes subscore | 23 (2.7) | 14 [ 10 – 24] |
| Perceptions subscore | 15 (2.8) | 15 [ 5 – 20] |
