# Supplementary material for: Physical activity prevalence and associated factors among Zimbabwean undergraduate students: A cross-sectional study
Source: PLOS Glob Public Health. 2025 Jul 9;5(7):e0004866. doi: 10.1371/journal.pgph.0004866 (PMC12240316; doi:10.1371/journal.pgph.0004866)
Supplement: S5 Table — (DOCX) [file pgph.0004866.s005.docx]

### **S5 Table: Factors associated with KAP crude odds ratios**

|  |  | **Knowledge** | **Attitudes** | **Perceptions** |
| --- | --- | --- | --- | --- |
| **Variable** | **Attribute** | **COR (95% CI)**, p**-value** | **COR (95% CI)**, p**-value** | **COR (95% CI)**, p**-value** |
| Gender | Male | 1.384 (1.088; 1.761), p =.008* | 1.194 (.947; 1.504), p = .287 | 1.194 (.947; 1.504), p = .134 |
|  | Female (Ref) |  |  |  |
| Institution | University A | 1.271 (.770; 2.099), p = .349 | 1.755 (1.074; 2.868), p =.025* | 1.755 (1.074; 2.868), p =.025* |
|  | University B | 1.437 (.941; 2.195), p = .093 | 1.335 (.884; 2.018). p = .170 | 1.840 (1.216; 2.784), p =.004* |
|  | University C (Ref) |  |  |  |
| Alcohol | No | 1.164 (.899; 1.506). p =.249 | 1.364 (1.068; 1.744), p =.013* | 1.445 (1.127; 1.853), p= .004* |
|  | Yes (Ref) |  |  |  |
| Smoking | No | 1.011 (.654; 1.562), p=.962 | 1.280 (.850; 1.927), p= .238 | 1.284 (.850; 1.941), p= .235 |
|  | Yes (Ref) |  |  |  |
| Drug /substance use | No | .831 (.474; 1.458), p=.519 | 1.108 (.664; 1.848), p =.696 | 1.011 (.599;1.709), p= .966 |
|  | Yes (Ref) |  |  |  |
| Faculty | Health | 1.107 (.849; 1.444), p=.452 | 1.008 (.783; 1.298), p=.952 | 1.196 (.926;1.545), p= .171 |
|  | Non-health (Ref) |  |  |  |
| Enrolment | Part-time | .750 (.410; 1.373), p =.352 | 1.139 (.626; 2.072) p =.670 | .735 (.407; 1.329), p=.308 |
|  | Full-time (Ref) |  |  |  |
| Study year | 1 | .943 (.681; 1.306), p = .723 |  |  |
|  | 2 | 1.361 (.995; 1.862), p = .054 |  |  |
|  | 3 | .840 (.584; 1.209). p =.248 |  |  |
|  | 4 & 5 (Ref) |  |  |  |
| Residence status | On campus | .831 (.623; 1.109), p = .208 | 1.181 (.900;1.551), p= .230 | .994 (.753;1.311), p=.966 |
|  | Within 5km | .950 (.697; 1.295), p =.747 | 1.103 (.827: 1.472), p= .504 | 1.096 (.815; 1.474), p =.543 |
|  | More than 5km (ref) |  |  |  |
| Sports participation | No | .223 (.135; .368), p= <.001* | .783 (.528; 1.162), p=.225 | .226 (.141; .362), p = <.001* |
|  | Sometimes | .531 (.322; .875), p =.013* | .825 (.562; 1.211), p=.325 | .475 (.298; .758), p =.002* |
|  | Daily (ref) |  |  |  |
| PA level | Low | .543 (.401; .737), p= <.001* | 1.027 (.769; 1.373), p=.855 | .602 (.448; .811), p=<.001* |
|  | Moderate | .788 (.594; 1.045), p=.098 | 1.244 (.958;1.616), p =.101 | .714 (.545; .934), p= .014* |
|  | High (ref) |  |  |  |
| Barriers | No | 1.282 (.992; 1.658). p=.058 | 1.882 (1.472;2.406), p=<.001* | 1.019 (.798; 1.300), p=.881 |
|  | Yes (ref) |  |  |  |
| Benefits | No | .308 (.240; .396), p= <.001* | .414 (.328; .522), p=<.001* | .262 (.205; .334), p<.001* |
|  | Yes (ref) |  |  |  |

** Denotes statistically significant variable, p< .05*
