# Supplementary material for: Physical activity prevalence and associated factors among Zimbabwean undergraduate students: A cross-sectional study
Source: PLOS Glob Public Health. 2025 Jul 9;5(7):e0004866. doi: 10.1371/journal.pgph.0004866 (PMC12240316; doi:10.1371/journal.pgph.0004866)
Supplement: S6 Table — (DOCX) [file pgph.0004866.s006.docx]

### **S6 Table: Associations of KAP; Adjusted odds ratios**

|  |  | **Knowledge** | **Attitudes** | **Perceptions** |
| --- | --- | --- | --- | --- |
| **Variable** | **Attribute** | **OR (95% CI), p-value** | **OR (95% CI), p-value** | **OR (95% CI), p-value** |
| Gender | Male | 1.024 (.786;1.335), p =.858 |  | 1.173 (.900;1.529), p=.239 |
|  | Female (ref) |  |  |  |
| Sports participation | No | 3.059 (1.808; 5.173), p=<.001* |  | 3.194 (1.921; 5.310), p= <.001* |
|  | Sometimes | 1.517 (.909; 2.533), p=.111 |  | 1.684 (1.032; 2.747), p=.037* |
|  | Daily (ref) |  |  |  |
| PA level | Low | 1.164 (.832; 1.629), p=.374 | .745 (.545; 1.019), p=.066 | 1.101 (.788; 1.539), p=.571* |
|  | Moderate | .985 (.729; 1.331), p=.922 | .684 (.518; .903), p=.007* | 1.173 (.874: 1.575), p=.287 |
|  | High (ref) |  |  |  |
| Barriers | No | .920 (.700: 1.210), p = .551 | .569 (.440; .734), p=<.001* | 1.259 (.964; 1.645), p=.091 |
|  | Yes (ref) |  |  |  |
| Benefits | No | 2.687 (2.063; 3.501), p=<.001* | 2.482 (1.941; 3.175), p=<.001 | 3.368 (2.591:4.378), p=<.001* |
|  | Yes (ref) |  |  |  |
| Institution | University A |  | .650 (.389; 1.086), p=.100 | .698 (.411; 1.184), p=.182 |
|  | University B |  | .869 (.559;1.351), p=.532 | .574 (.329; 1.001), p=.051 |
|  | University C (Ref) |  |  |  |
| Alcohol | No |  | .809 (.624; 1.048), p=.109 | .629 (.478; .827), p=<.001* |
|  | Yes (ref) |  |  |  |
| Year of study | 1 |  | .673 (.478; .949), P=.024* |  |
|  | 2 |  | .993 (.732; 1.347), P=.964 |  |
|  | 3 |  | 1.066 (.739; 1.539), P=.732 |  |
|  | 4&5 (ref) |  |  |  |
| Faculty | Health |  |  | 1.199 (.767; 1.873), p=.425 |
|  | Non-health (ref) |  |  |  |
